# Tofogliflozin long-term effects on atherosclerosis progression and major clinical parameters in patients with type 2 diabetes mellitus lacking a history of cardiovascular disease: a 2-year extension study of the UTOPIA trial

**DOI:** 10.1186/s12933-023-01879-4

**Published:** 2023-06-22

**Authors:** Naoto Katakami, Tomoya Mita, Hidenori Yoshii, Toshihiko Shiraiwa, Tetsuyuki Yasuda, Yosuke Okada, Akira Kurozumi, Masahiro Hatazaki, Hideaki Kaneto, Takeshi Osonoi, Tsunehiko Yamamoto, Nobuichi Kuribayashi, Kazuhisa Maeda, Hiroki Yokoyama, Keisuke Kosugi, Kentaro Ohtoshi, Isao Hayashi, Satoru Sumitani, Mamiko Tsugawa, Kayoko Ryomoto, Ken Kato, Tadashi Nakamura, Satoshi Kawashima, Yasunori Sato, Hirotaka Watada, Iichiro Shimomura, the UTOPIA study investigators, K. Komiyama, T. Shimizu, S. Kamei, T. Kinoshita, M. Shimoda, M. Saito, N. Fujiki, Y. Fujita, S. Shimizu, Y. Umayahara, Y. Irie, R. Kataoka, Y. Kiyohara, M. Ohashi, K. Ryomoto, Y. Takahi, Y. Fujishima, Y. Fujita, A. Fukuhara, K. Fukui, Y. Hosokawa, A. Imagawa, H. Iwahashi, K. Mukai, T. Katsura, D. Kawamori, T. Kimura, S. Kobayashi, J. Kozawa, F. Kubo, N. Maeda, T. Matsuoka, K. Miyashita, S. Nakata, H. Ninomiya, H. Nishizawa, Y. Okuno, M. Otsuki, F. Sakamoto, S. Sasaki, I. Sato, N. Shimo, I. Shimomura, M. Takahara, T. Takano, A. Tokunaga, S. Uno, M. Yamaoka, S. Yoneda, M. Hajime, K. Koikawa, F. Kuno, K. Matsushita, M. Narisawa, K. Tanaka, K. Sugai, K. Torimoto

**Affiliations:** 1 Department of Metabolic Medicine, Osaka University Graduate School of Medicine, 2-2, Yamadaoka, Suita, Osaka 565-0871 Japan; 2 Department of Metabolism and Atherosclerosis, Osaka University Graduate School of Medicine, 2-2, Yamadaoka, Suita, Osaka 565-0871 Japan; 3 Department of Metabolism & Endocrinology, Juntendo University Graduate School of Medicine, Hongo 2-1-1, Bunkyo-ku, Tokyo, 113-8421 Japan; 4 Department of Medicine, Diabetology & Endocrinology, Juntendo Tokyo Koto Geriatric Medical Center, Koto-ku, Tokyo, 136-0075 Japan; 5 Shiraiwa Medical Clinic, 4-10-24 Hozenji, Kashiwara, Osaka 582-0005 Japan; 6 Department of Endocrinology and Metabolism, Osaka Police Hospital, 10-31, Kitayama-Cho, Tennoji-ku, Osaka, 543-0035 Japan; 7 First Department of Internal Medicine, School of Medicine, University of Occupational and Environmental Health, 1-1, Iseigaoka, Yahatanishi-ku, Kitakyushu, 807-8555 Japan; 8 Department of Diabetes and Endocrinology, Osaka General Medical Center, 3-1-56, Bandai-Higashi, Sumiyoshi-ku, Osaka, 558-8558 Japan; 9 Department of Diabetes, Endocrinology and Metabolism, Kawasaki Medical School, 577 Matsushima, Kurashiki, Okayama 701-0192 Japan; 10 Nakakinen Clinic, 745-5, Nakadai, Naka, Ibaraki 311-0113 Japan; 11 Diabetes and Endocrinology, Kansai Rosai Hospital, 3-1-69, Inabaso, Amagasaki, Hyogo Japan; 12 Misaki Naika Clinic, 6-44-9, Futawa-Higashi, Funabashi, Chiba Japan; 13 Kitasenri Maeda Clinic, 4-119, Furuedai, Suita, Osaka 565-0874 Japan; 14 Jiyugaoka Medical Clinic, West 6, South 6-4-3, Obihiro, Hokkaido 080-0016 Japan; 15 Kosugi Medical Clinic, 3-9, Tamatsukurimoto-Cho, Tennoji-ku, Osaka, 543-0014 Japan; 16 Otoshi Medical Clinic, 8-47, KakudachoOsaka Kita-ku, Osaka, 530-0017 Japan; 17 Hayashi Clinic, 3-9-23, Koshienguchi, Nishinomiya, Hyogo 663-8113 Japan; 18 Center for Diabetes and Endocrinology, Nippon Life Hospital, 2-1-54 Enokojima, Nishi-ku, Osaka, 550-0006 Japan; 19 Department of Endocrinology and Metabolism, Ikeda Municipal Hospital, 3-1-18, Jonan, Ikeda, Osaka 563-8510 Japan; 20 Center for Diabetes Mellitus, Osaka Rosai Hospital, 1179-3 Nagasone-Cho, Kita-ku, Sakai, Osaka 591-8025 Japan; 21 Diabetes Center, National Hospital Organization Osaka National Hospital, 2-1-14, Hoenzaka, Chuo-ku, Osaka, 540-0006 Japan; 22 Department of Internal Medicine, Kawasaki Hospital, 3-3-1, Higashiyamacho, Kobe Hyogo-ku, Hyogo, 652-0042 Japan; 23 Kanda Naika Clinic, 5-21-3, Hannancho, Osaka Abeno-ku, Osaka, 545-0021 Japan; 24 Department of Preventive Medicine and Public Health, Keio University School of Medicine, 45 Shinanomachi Shinjuku-ku, Tokyo, 160-8582 Japan; 25 Present Address: Department of Diabetology and Endocrinology, Pref Osaka Saiseikai Izuo Hospital, 3-4-5 Kitamura, Taisho, Osaka 551-0032 Japan; 26 Present Address: Department of Diabetes and Endocrinology, Meiwa Hospital, 4-31 Agenaruo, Nishinomiya, Hyogo 663-8186 Japan

**Keywords:** Atherosclerosis, Brachial-ankle pulse wave velocity, Cardiovascular risk factors, Carotid intima-media thickness, Sodium-glucose cotransporter 2 inhibitor, Tofogliflozin

## Abstract

**Background:**

This study aimed to assess the long-term effects of tofogliflozin, a sodium-glucose cotransporter 2 (SGLT2) inhibitor, on atherosclerosis progression and major clinical parameters in patients with type 2 diabetes lacking an apparent history of cardiovascular disease.

**Methods:**

This was a prospective observational 2-year extension study of the “Using TOfogliflozin for Possible better Intervention against Atherosclerosis for type 2 diabetes patients (UTOPIA)” trial, a 2-year randomized intervention study. The primary endpoints represented changes in the carotid intima-media thickness (IMT). Secondary endpoints included brachial-ankle pulse wave velocity (baPWV) and biomarkers for glucose metabolism, lipid metabolism, renal function, and cardiovascular risks.

**Results:**

The mean IMT of the common carotid artery (IMT-CCA) significantly decreased in both the tofogliflozin (− 0.067 mm, standard error 0.009, p < 0.001) and conventional treatment groups (− 0.080 mm, SE 0.009, p < 0.001) throughout the follow-up period; however, no significant intergroup differences in the changes (0.013 mm, 95% confidence interval (CI) − 0.012 to 0.037, p = 0.32) were observed in a mixed-effects model for repeated measures. baPWV significantly increased in the conventional treatment group (82.7 ± 210.3 cm/s, p = 0.008) but not in the tofogliflozin group (− 17.5 ± 221.3 cm/s, p = 0.54), resulting in a significant intergroup difference in changes (− 100.2 cm/s, 95% CI − 182.8 to − 17.5, p = 0.018). Compared to the conventional treatment group, tofogliflozin significantly improved the hemoglobin A1c and high-density lipoprotein cholesterol levels, body mass index, abdominal circumference, and systolic blood pressure. The frequencies of total and serious adverse events did not vary significantly between the groups.

**Conclusions:**

Tofogliflozin was not associated with improved inhibition of carotid wall thickening but exerted long-term positive effects on various cardiovascular risk factors and baPWV while showing a good safety profile.

**Supplementary Information:**

The online version contains supplementary material available at 10.1186/s12933-023-01879-4.

## Background

Sodium-glucose cotransporter 2 (SGLT2) inhibitors lower blood glucose by promoting urinary glucose excretion in an insulin-independent manner. Hence, SGLT2 inhibitors are associated with a lower risk of hypoglycemia than conventional anti-diabetic agents. SGLT2 inhibitors also have pleiotropic effects, such as reducing body weight [1], visceral adipose tissue [2], and blood pressure [3]; improving lipid profile [4]; renal protection [5]; and remarkable cardiovascular protection [6, 7].

Tofogliflozin is an SGLT2 inhibitor with high selectivity for SGLT2. Suzuki et al. measured the sodium-dependent glucose uptake in SGLT2-overexpressing cells and evaluated SGLT2 selectivity against SGLT1, expressed as the ratio of inhibitory concentration 50 (IC50) for each compound. Their study showed that tofogliflozin (2900-fold) was highly selective compared to dapagliflozin (610-fold), canagliflozin (290-fold), ipragliflozin (860-fold), empagliflozin (1100-fold), and luseogliflozin (1600-fold) [8]. Tofogliflozin also has a short elimination half-life (5–6 h) with a high level of urinary excretion [9], which reduces the risk of nocturnal hypoglycemia [10]. In addition to its effect on glycemic control, tofogliflozin improves high-density lipoprotein cholesterol (HDL-C) and triglyceride (TG) levels, decreases body weight and blood pressure, and elevates circulating adiponectin levels [11]. We previously investigated the effect of tofogliflozin on carotid artery intima-media thickness (IMT) [12], which is a marker of atherosclerosis [13], arterial stiffness [14], and patients’ quality of life (QOL) [15], in the “Using TOfogliflozin for Possible better Intervention against Atherosclerosis for type 2 diabetes patients (UTOPIA)” trial. Although tofogliflozin did not delay the progression of IMT thickening, it significantly improved glycemic control, body weight, body mass index (BMI), abdominal circumference, systolic blood pressure, HDL-C, arterial stiffness, and treatment-related QOL [12, 14, 15] during the 2-year intervention period.

In several long-term observational follow-up studies after randomized controlled trials (RCTs) of anti-diabetic agents, it has been shown that emergent risk reductions for diabetic complications were observed during the post-trial follow-up period, highlighting the benefits of early and intensive glycemic control [16]. However, the long-term effects of tofogliflozin on atherosclerosis progression in patients with type 2 diabetes lacking an apparent history of cardiovascular disease have not been investigated. In this prospective observational 2-year extension study of the UTOPIA trial, we aimed to assess the long-term effects of tofogliflozin on the progression of atherosclerosis and major clinical parameters in patients with type 2 diabetes mellitus without an apparent history of cardiovascular disease.

## Methods

### Study design

This was a prospective observational 2-year extension study of the UTOPIA trial [17], which was conducted at 22 medical institutions in Japan (Additional file 1). The main UTOPIA study and this extension study were registered in the University Hospital Medical Information Network Clinical Trials Registry (UMIN-CTR), a non-profit organization in Japan, and met the requirements of the International Committee of Medical Journal Editors (UMIN000017607 and UMIN000032601, respectively). Patient enrollment was conducted between January 2018 and April 2019, and each enrolled patient was observed for 2 years. The study protocol was approved by the Ethical Review Board of Osaka University Hospital and the institutional review board of each participating center according to the Ethical Guidelines for Medical and Health Research Involving Human Subjects issued by the Ministry of Health, Labour and Welfare in Japan. The study was conducted in accordance with the Declaration of Helsinki, the Ethical Guidelines for Medical and Health Research Involving Human Subjects, and other current legal regulations in Japan. To avoid bias, data management and monitoring were conducted by a third-party entity (Soiken Inc., Toyonaka, Osaka, Japan). All the authors vouch for the completeness and accuracy of the data and the analyses and for the fidelity of the trial to the protocol.

### Study population

Japanese subjects with type 2 diabetes mellitus who had participated in the main UTOPIA study were asked to participate in this extension study. The sample size of the main UTOPIA study was calculated as follows: the increase in carotid IMT in diabetic patients is estimated to be 0.034 ± 0.054 mm/year (mean ± standard deviation [SD]), and a 1% improvement in hemoglobin A1c (HbA1c) levels improves IMT by 0.02 mm/year [18]. Therefore, to obtain 90% power to detect a difference of 0.04 mm in IMT between the two groups during the 2-year observation period, with an SD of 0.108 mm for the individual difference estimated to be common to both groups and a significance level of 0.05, at least 155 patients in each group must be enrolled. The dropout rate during the 2-year observation period was assumed to be 10%, and the target number of patients to be enrolled was 340 (170 in each group) [17]. As the number of subjects in the Extension study was reduced to 146 in the tofogliflozin group and 145 in the conventional treatment group, the power to detect a difference of 0.04 mm between the groups under the condition of α error = 0.05 was calculated to be 0.88.

The inclusion criterion for this extension study was as follows: subjects who had participated in the main UTOPIA study and provided written informed consent to participate in this extension study after a full explanation of the extension study. The exclusion criterion for this extension study was as follows: subjects deemed unsuitable for participation in this extension study by the investigators. The inclusion criteria for the main study were as follows: (1) Japanese patients with type 2 diabetes mellitus who are not achieving the glycemic control goals described in the “Treatment Guide for Diabetes 2014–2015” edited by the Japan Diabetes Society [19] and have an HbA1c level of < 9% (according to this guide, if it can be achieved with appropriate diet and exercise alone or without side effects such as hypoglycemia while on drug therapy, the target HbA1c level should be < 6%, otherwise < 7% was set to the target value, respectively); (2) no changes (including new prescriptions) in their antidiabetic, antithrombotic, or antihypertensive medications or therapeutic agents for dyslipidemia for at least 12 weeks before signing the consent form; (3) age 30–74 at the time of giving consent; and (4) ability to provide informed consent. The exclusion criteria for the main study were as follows: (1) type 1 or secondary diabetes; (2) in the perioperative period or having a serious infection or injury; (3) a history of myocardial infarction, angina, stroke, or cerebral infarction; (4) severe renal dysfunction or end-stage renal failure (estimated glomerular filtration rate (eGFR) < 30 mL/min/1.73 m^2^); (5) serious liver function impairment (aspartate aminotransferase ≥ 100 U/L); (6) moderate to severe heart failure (class 3 or worse based on the New York Heart Association Functional Classification); (7) urinary tract or genital infection; (8) pregnancy, possible pregnancy, nursing, or planning to conceive a child; (9) history of hypersensitivity to the study drug; (10) present or past history of a malignant tumor (exceptions: patients not on medication for a malignant tumor with no recurrence of the disease thus far and no recurrence risks during the study were allowed to participate); (11) prohibited from using tofogliflozin; or (12) other ineligibility determined by an investigator.

### Randomization and study intervention

As this was a prospective observational extension study of the UTOPIA trial, no randomization was conducted, and no drug or treatment was prohibited or restricted in this study. In the main UTOPIA study, the enrolled subjects were randomly assigned to a tofogliflozin treatment group or a conventional treatment group without SGLT2 inhibitors. In the tofogliflozin group, 20 mg of tofogliflozin once daily was started in addition to the ongoing therapy. However, the addition of an alternative antidiabetic agent (excluding another SGLT2 inhibitor) was permitted 12 weeks after randomization. In the conventional treatment group, either the dosage of the ongoing therapy was increased, or a concomitant oral glucose-lowering drug (excluding any other SGLT2 inhibitor) was added 12 weeks after randomization. Treatment was continued to achieve the target value specified in the Japanese Treatment Guide for Diabetes [19] (generally an HbA1c level < 7.0%) in both groups. In case of hypoglycemia, the dosage of the concomitant oral glucose-lowering drug was titrated.

### Observation items and schedule

The enrolled subjects were observed for 104 weeks in the main study followed by additional 104 weeks in this extension study, totaling 208 weeks. In the main study, clinical and biochemical data were collected at 0, 26, 52, 78, and 104 weeks after randomization, and in this extension study, the data were collected at 156 and 208 weeks after the randomization in the main study.

### Study outcomes

The primary endpoints in this extension study were the changes in the mean and maximum carotid intima-media thickness of the common carotid artery (IMT-CCA) measured by carotid arterial echography from the baseline (week 0) in the main study to week 208. In particular, the change in the mean of the right and left mean IMT-CCA from baseline to week 208 was considered the most important endpoint in this study. Secondary endpoints included changes in biomarkers of glycemic control (HbA1c and fasting blood glucose), lipids (total cholesterol (TC), HDL-C, TG, and low-density lipoprotein cholesterol (LDL-C)), renal function (serum creatinine, urinary albumin excretion (UAE), and eGFR), and arteriosclerosis and cardiovascular risks (brachial-ankle pulse wave velocity (baPWV) and ankle-brachial index (ABI)), the frequency of cardiovascular events including ischemic heart disease (sudden cardiac death, acute myocardial infarction, unstable angina, and undergoing coronary revascularization), cerebrovascular disorders (cerebral infarction, intracerebral hemorrhage, and subarachnoid hemorrhage), and peripheral arterial disease (arteriosclerosis obliterans and leg amputation), and the frequency of adverse events.

### Measurement of carotid IMT

Ultrasonography scans of the carotid artery were performed by expert, specifically-trained sonographers based on the Japan Society of Ultrasonics in Medicine guidelines [20]. To avoid inter-sonographer variability, each participant was examined by the same sonographer with the same equipment (high-resolution B-mode ultrasound scanner equipped with a high-frequency [> 7.5 MHz] linear transducer with a limit of detection of < 0.1 mm) throughout all the visits. Scanning of the extracranial carotid artery was performed bilaterally in different longitudinal and transverse projections, and the site of greatest thickness, including plaque lesions, was sought along the arterial walls. The IMT was measured as the distance between two parallel echogenic lines corresponding to the vascular lumen and the adventitial layer.

To avoid inter-reader variability, all scans were stored electronically and sent to the IMT evaluation committee. The sent images were inspected in random order by experienced investigators who were not informed of the clinical characteristics of the subjects, using automated digital edge detection software (Intimascope; MediaCross, Tokyo, Japan) [21]. The software system averaged approximately 200 points of the IMT values in the segment 2 cm proximal to the dilation of the carotid sinus (mean-IMT-CCA). In addition, the maximum thicknesses of the intima and media layers, including the plaque lesions, in the common carotid arteries (max-IMT-CCA) were captured separately. The same systematic procedures for analyzing carotid IMT were used in our previous studies [22, 23]. Reproducibility analysis of replicate measurements in the randomly selected 20 subjects yielded absolute mean differences of 0.02 ± 0.01 and 0.01 ± 0.01 for mean-IMT-CCA and max-IMT-CCA, respectively. The intra-observer coefficients of variation for the measurements of mean-IMT-CCA and max-IMT-CCA were 1.1% and 0.7%, respectively.

### Statistical analysis

Analyses of the primary and secondary endpoints were performed using data from the full analysis set (FAS) population, including all subjects enrolled in this extension study, and excluding those violating the protocol severely (without written consent, enrollment outside the contract period, etc.). In principle, missing values were not complemented.

Primary analysis was performed using the mixed-effects model for repeated measures (MMRM) with the treatment group, time (week), and interactions between the treatment group and time (week) as fixed effects, and subjects nested by group as random effects. An unstructured covariate was used to model the covariance of within-subject variability. The Kenward-Roger method was used to estimate the degrees of freedom. The sensitivity analysis assessed differences in changes in IMT-CCA from baseline between the two groups using analysis of covariance (ANCOVA) models that included the treatment group, age, sex, baseline IMT-CCA, systolic blood pressure, and administration of statins. For the occurrence of cardiovascular events as a secondary outcome, the time to onset was analyzed using the log-rank test and Cox proportional hazard model.

Baseline and follow-up group comparisons were performed using Student’s t-test or Wilcoxon rank-sum test for continuous variables and Fisher’s exact test or chi-square test for categorical variables. Changes from baseline to treatment visits were assessed using a paired t-test and Wilcoxon signed-rank test within the group. The frequency and proportion of patients reporting AEs were derived from each treatment group and compared using Fisher’s exact test.

All p values were two-sided. Statistical significance was set at p < 0.05. All analyses were performed using SAS version 9.4 (SAS Institute, Inc., Cary, NC, USA). All statistical analyses were performed by a third-party entity (Soiken Inc.) under the supervision of an independent biostatistician. The corresponding author had full access to all data in the study and had final responsibility for the decision to submit for publication.

## Results

### Allocation and patient characteristics

**Fig. 1:**
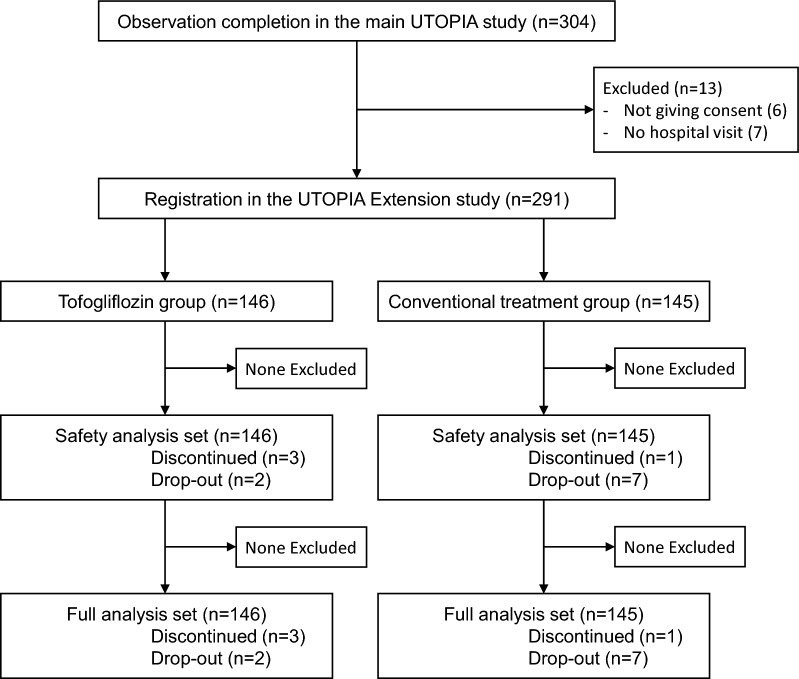
Study flow chart showing patient enrollment and analysis

In the main UTOPIA study, 340 participants were randomly allocated to either the tofogliflozin group (n = 169) or the conventional treatment group (n = 171) between January 2016 and November 2016. Of these, 304 completed the follow-up. After the completion of the follow-up in the main UTOPIA study, between June 2018 and April 2019, each participant was asked to participate in this UTOPIA Extension study. Since 13 participants were excluded (6 participants did not give consent to participate in this UTOPIA Extension study, and 7 participants discontinued the hospital visits), 291 participants were enrolled in this UTOPIA Extension study. Of these, 146 and 145 were assigned to the tofogliflozin and conventional treatment groups, respectively, in the UTOPIA Extension study (Fig. 1). The characteristics of patients who participated in the UTOPIA Extension study, as recorded at week 0 (baseline) of the main UTOPIA study, were well balanced between the two groups, except for the use of angiotensin II receptor blockers and eicosapentaenoic acid (Table 1).

**Table 1:** Clinical characteristics of patients at week 0 (baseline) of the main UTOPIA study

Parameters	Tofogliflozin group	Conventional treatment group	intergroup p value
Sex (males)	87 (59.6)	88 (60.7)	0.90
Age (years)	61.8 ± 8.9	61.0 ± 9.5	0.47
Current smoking	30 (20.7)	25 (17.2)	0.55
Hypertension	77 (52.7)	91 (62.8)	0.10
Dyslipidemia	91 (62.3)	102 (70.3)	0.17
Duration of diabetes (years)	12.0 ± 8.1	12.4 ± 8.2	0.71
Diabetic retinopathy	26 (18.1)	28 (19.4)	0.88
Diabetic nephropathy	42 (28.8)	46 (31.7)	0.61
Use of glucose-lowering agent	133 (91.1)	129 (89.0)	0.56
Metformin	78 (53.4)	88 (60.7)	0.24
Sulfonylurea	35 (24.0)	39 (26.9)	0.59
Glinides	8 (5.5)	9 (6.2)	0.81
Thiazolidinediones	17 (11.6)	19 (13.1)	0.73
α-Glucosidase inhibitor	22 (15.1)	21 (14.5)	1.00
DPP-4 inhibitor	68 (46.6)	82 (56.6)	0.10
GLP-1 receptor agonist	19 (13.0)	10 (6.9)	0.12
Insulin	27 (18.5)	32 (22.1)	0.47
Use of antihypertensive drug	69 (47.3)	84 (57.9)	0.08
Angiotensin-converting enzyme inhibitor	2 (1.4)	5 (3.4)	0.28
Angiotensin II receptor blocker	55 (37.7)	73 (50.3)	0.034
Direct renin inhibitor	2 (1.4)	0 (0.0)	0.50
Calcium channel blocker	43 (29.5)	48 (33.1)	0.53
Diuretic drug	7 (4.8)	14 (9.7)	0.12
α-Adrenergic receptor antagonist	1 (0.7)	0 (0.0)	1.00
β-Adrenergic receptor antagonist	3 (2.1)	2 (1.4)	1.00
α1β-Adrenergic Receptor Blocker	3 (2.1)	10 (6.9)	0.05
Others	0 (0.0)	0 (0.0)	–
Use of lipid-lowering agent	72 (49.3)	87 (60.0)	0.08
Statin	63 (43.2)	74 (51.0)	0.20
Ezetimibe	10 (6.8)	11 (7.6)	0.83
Resin	0 (0.0)	0 (0.0)	–
Fibrates	8 (5.5)	5 (3.4)	0.57
Eicosapentaenoic acid	0 (0.0)	5 (3.4)	0.030
Eicosapentaenoic acid/docosahexaenoic acid	2 (1.4)	2 (1.4)	1.00
Others	0 (0.0)	0 (0.0)	–
Use of antithrombotic agent	14 (9.6)	14 (9.7)	1.00
Antiplatelet agent	12 (8.2)	10 (6.9)	0.83
Anticoagulant	2 (1.4)	4 (2.8)	0.45
Others	0 (0.0)	0 (0.0)	–

Data are presented as number (%) of patients or mean ± standard deviation

### Biomarkers for arteriosclerosis

The common mean-IMT-CCA, right max-IMT-CCA, and left max-IMT-CCA at baseline in the tofogliflozin conventional treatment groups are shown in Table 2. Plaques and/or thickened (focal IMT ≥ 1.0 mm) lesions were observed in the right CCA in 208 patients (99 and 109 in the tofogliflozin and conventional treatment groups, respectively), in the left CCA in 218 patients (107 and 111 in the tofogliflozin and conventional treatment groups, respectively), and bilaterally in 147 patients (65 and 82 in the tofogliflozin and conventional treatment groups, respectively).

**Table 2:** Effects of tofogliflozin on biomarkers of arteriosclerosis

	Observation point	Tofogliflozin group		Conventional treatment group		Intergroup p value for measurements	Treatment effect; mean intergroup difference in change (95% CI) (intergroup p value)
		Measurement	Mean change (intragroup p value)	Measurement	Mean change (intragroup p value)		
Common mean IMT-CCA (mm)	Week 0	0.873 ± 0.156 (n = 146)		0.870 ± 0.159 (n = 145)		0.89	
	Week 104	0.738 ± 0.145 (n = 145)	− 0.135 (0.007) (p < 0.001)	0.724 ± 0.136 (n = 143)	− 0.145 (0.007) (p < 0.001)	0.38	0.010 (− 0.010, 0.030) (p = 0.32)
	Week 208	0.807 ± 0.149 (n = 137)	− 0.067 (0.009) (p < 0.001)	0.790 ± 0.141 (n = 129)	− 0.080 (0.009) (p < 0.001)	0.33	0.013 (− 0.012, 0.037) (p = 0.32)
Right maximum IMT-CCA (mm)	Week 0	1.064 ± 0.300 (n = 146)		1.069 ± 0.263 (n = 144)		0.88	
	Week 104	0.911 ± 0.305 (n = 145)	− 0.154 (0.013) (p < 0.001)	0.879 ± 0.218 (n = 142)	− 0.188 (0.013) (p < 0.001)	0.32	0.034 (− 0.002, 0.069) (p = 0.06)
	Week 208	1.031 ± 0.369 (n = 137)	− 0.040 (0.023) (p = 0.09)	1.012 ± 0.338 (n = 129)	− 0.052 (0.024) (p = 0.031)	0.67	0.012 (− 0.053, 0.078) (p = 0.72)
Left maximum IMT-CCA (mm)	Week 0	1.148 ± 0.383 (n = 146)		1.127 ± 0.384 (n = 144)		0.65	
	Week 104	0.965 ± 0.391 (n = 145)	− 0.185 (0.021) (p < 0.001)	0.929 ± 0.313 (n = 142)	− 0.196 (0.021) (p < 0.001)	0.39	0.012 (− 0.048, 0.071) (p = 0.70)
	Week 208	1.099 ± 0.413 (n = 137)	− 0.053 (0.029) (p = 0.07)	1.096 ± 0.503 (n = 129)	− 0.048 (0.029) (p = 0.10)	0.96	− 0.005 (− 0.086, 0.076) (p = 0.91)
Mean baPWV (cm/s)	Week 0	1706.2 ± 419.5 (n = 75)		1655.2 ± 319.5 (n = 65)		0.42	
	Week 104	1685.5 ± 352.3 (n = 72)	− 54.5 ± 235.9 (p = 0.06)	1738.6 ± 362.2 (n = 63)	56.5 ± 199.1 (p = 0.033)	0.39	− 111.0 (− 188.6, − 33.5) (p = 0.005)
	Week 208	1689.6 ± 391.2 (n = 65)	− 17.5 ± 221.3 (p = 0.54)	1774.4 ± 329.4 (n = 53)	82.7 ± 210.3 (p = 0.008)	0.21	− 100.2 (− 182.8, − 17.5) (p = 0.018)
Mean ABI	Week 0	1.13 ± 0.10 (n = 104)		1.14 ± 0.08 (n = 95)		0.34	
	Week 104	1.14 ± 0.08 (n = 97)	0.01 ± 0.09 (p = 0.30)	1.15 ± 0.08 (n = 88)	0.00 ± 0.08 (p = 0.98)	0.65	0.01 (− 0.02, 0.04) (p = 0.47)
	Week 208	1.15 ± 0.09 (n = 88)	0.01 ± 0.09 (p = 0.17)	1.14 ± 0.10 (n = 73)	0.00 ± 0.09 (p = 0.68)	0.61	0.02 (− 0.01, 0.05) (p = 0.22)

Data are presented as the mean ± standard deviation or adjusted mean change (standard error). Comparisons of the IMT-CCA, baPWV, and ABI values during treatment with those at baseline were performed using a one-sample t-test based on the mixed-effects model for repeated measures. Intergroup differences in IMT-CCA measurements were analyzed using Student’s t-test. Intergroup differences in changes in IMT-CCA from baseline (treatment effect) were analyzed using the mixed-effects model for repeated measures. The treatment group, week, interactions between treatment group and week, age, sex, use of insulin at baseline, and baseline IMT-CCA were included as fixed effects. IMT-CCA: intima-media thickness of the common carotid artery; baPWV: brachial-ankle pulse wave velocity; ABI: ankle-brachial index; 95% CI: 95% confidence interval

During the main UTOPIA study (from baseline to week 104), the common mean-IMT-CCA and right and left max-IMT-CCA significantly decreased in both the tofogliflozin and conventional treatment groups, although no significant intergroup differences in the changes were observed in a mixed-effects model for repeated measures. The common mean-IMT-CCA showed a significant reduction throughout the follow-up period (from baseline to the end of the UTOPIA Extension study in both groups (week 208)); however, no significant intergroup differences in the changes were observed (Table 2). In the tofogliflozin group, both the right and left max-IMT-CCA showed a tendency to decrease throughout the follow-up period, but the difference was not statistically significant (p = 0.09 and p = 0.07, respectively). In the conventional treatment group, the right max-IMT-CCA significantly decreased throughout the follow-up period; however, the significant reduction in the left max-IMT-CCA at week 104 disappeared at week 208. No significant intergroup differences in the changes were observed in the right and left max-IMT-CCA throughout the follow-up period. Moreover, ANCOVA models that included treatment group, age, sex, use of insulin, baseline IMT, systolic blood pressure, and administration of statins produced findings that resembled those generated by the mixed-effects models (data not shown).

The mean baPWV significantly increased in the conventional treatment group throughout the follow-up period and was maintained or even decreased in the tofogliflozin group, resulting in a significant intergroup difference in the changes throughout the follow-up period (Table 2).

The ABI did not change significantly throughout the follow-up period in either group. When the ABI was classified into three categories (abnormally low (ABI ≤ 0.9), within the normal range (0.9 < ABI < 1.3), and abnormally high (1.3 ≤ ABI)), the proportion of patients whose ABI was within the normal range was as high as more than 95% at baseline, and no significant intergroup differences were observed throughout the follow-up period (Additional file 2).

### Glycemic control

During the main UTOPIA study, HbA1c and fasting blood glucose levels significantly decreased in the tofogliflozin group but not in the conventional treatment group, resulting in a significant intergroup difference in changes. Significant improvements in HbA1c and fasting blood levels were maintained throughout the follow-up period in the tofogliflozin group (Table 3). Significant intergroup differences in the changes in HbA1c levels were also maintained throughout the follow-up period. There was no difference in the proportion of patients treated with other glucose-lowering agents throughout the observation period; however, the tofogliflozin group tended to have fewer patients treated with biguanides and DPP-4 inhibitors than did the conventional treatment group (Additional file 3).

**Table 3:** Effects of tofogliflozin on clinical laboratory tests

	Observation point	Tofogliflozin group		Conventional treatment group		Intergroup p value for measurements	Treatment effect; mean intergroup difference in change (95% CI) (intergroup p value)
		Measurement	Change from baseline (intragroup p value)	Measurement	Change from baseline (intragroup p value)		
Body Mass Index (kg/m^2^)	Week 0	26.7 ± 5.4 (n = 146)		26.7 ± 4.4 (n = 144)		0.99	
	Week 104	25.8 ± 5.5 (n = 145)	− 0.9 ± 1.4 (p < 0.001)	26.6 ± 4.2 (n = 144)	− 0.2 ± 1.8 (p = 0.21)	0.20	− 0.7 (− 1.1, − 0.4) (p < 0.001)
	Week 208	25.5 ± 4.9 (n = 143)	− 1.2 ± 2.3 (p < 0.001)	26.1 ± 4.0 (n = 137)	− 0.6 ± 2.1 (p = 0.001)	0.28	− 0.6 (− 1.2, − 0.1) (p = 0.021)
Waist circumference (cm)	Week 0	92.5 ± 12.1 (n = 131)		93.1 ± 11.2 (n = 130)		0.71	
	Week 104	91.8 ± 13.3 (n = 126)	− 1.2 ± 6.1 (p = 0.039)	94.3 ± 10.8 (n = 129)	1.4 ± 4.3 (p < 0.001)	0.11	− 2.6 (− 3.9, − 1.2) (p < 0.001)
	Week 208	90.5 ± 11.9 (n = 108)	− 1.5 ± 6.0 (p = 0.011)	93.4 ± 9.6 (n = 111)	0.4 ± 5.1 (p = 0.48)	0.05	− 1.9 (− 3.4, − 0.3) (p = 0.016)
HbA1c (%)	Week 0	7.4 ± 0.7 (n = 146)		7.3 ± 0.7 (n = 145)		0.09	
	Week 104	7.1 ± 0.7 (n = 146)	− 0.3 ± 0.8 (p < 0.001)	7.3 ± 0.9 (n = 144)	0.1 ± 0.7 (p = 0.24)	0.006	− 0.4 (− 0.6, − 0.2) (p < 0.001)
	Week 208	7.2 ± 0.8 (n = 145)	− 0.2 ± 0.8 (p = 0.001)	7.3 ± 0.8 (n = 140)	0.0 ± 0.8 (p = 0.97)	0.48	− 0.2 (− 0.4, 0.0) (p = 0.019)
HbA1c (mmol/mol)	Week 0	57.6 ± 7.9 (n = 146)		56.0 ± 7.7 (n = 145)		0.09	
	Week 104	53.8 ± 8.0 (n = 146)	− 3.8 ± 8.2 (p < 0.001)	56.8 ± 10.2 (n = 144)	0.7 ± 7.4 (p = 0.24)	0.006	− 4.5 (− 6.3, − 2.7) (p < 0.001)
	Week 208	55.1 ± 8.5 (n = 145)	− 2.5 ± 9.1 (p = 0.001)	55.9 ± 8.9 (n = 140)	0.0 ± 8.5 (p = 0.97)	0.48	− 2.5 (− 4.5, − 0.4) (p = 0.019)
Fasting blood glucose (mmol/L)	Week 0	7.9 ± 1.8 (n = 146)		7.8 ± 1.9 (n = 145)		0.86	
	Week 104	7.2 ± 1.5 (n = 140)	− 0.7 ± 1.8 (p < 0.001)	8.0 ± 2.0 (n = 141)	0.1 ± 1.8 (p = 0.41)	< 0.001	− 0.8 (− 1.3, − 0.4) (p < 0.001)
	Week 208	7.5 ± 1.8 (n = 131)	− 0.4 ± 2.2 (p = 0.033)	7.9 ± 2.3 (n = 126)	0.0 ± 2.5 (p = 0.92)	0.11	− 0.4 (− 1.0, 0.2) (p = 0.18)
Serum creatinine (mg/dL)	Week 0	0.73 ± 0.20 (n = 145)		0.73 ± 0.20 (n = 144)		0.96	
	Week 104	0.75 ± 0.22 (n = 146)	0.02 ± 0.11 (p = 0.012)	0.76 ± 0.23 (n = 145)	0.03 ± 0.10 (p = 0.001)	0.77	− 0.01 (− 0.03, 0.02) (p = 0.61)
	Week 208	0.77 ± 0.22 (n = 143)	0.05 ± 0.12 (p < 0.001)	0.82 ± 0.43 (n = 137)	0.10 ± 0.32 (p < 0.001)	0.17	− 0.05 (− 0.11, 0.00) (p = 0.07)
UAE (ln(mg/g・Cr))	Week 0	2.86 ± 1.41 (n = 138)		3.10 ± 1.61 (n = 134)		0.19	
	Week 104	2.76 ± 1.48 (n = 145)	− 0.09 ± 1.00 (p = 0.31)	3.20 ± 1.67 (n = 140)	0.14 ± 0.85 (p = 0.07)	0.019	− 0.22 (− 0.44, 0.00) (p = 0.05)
	Week 208	3.00 ± 1.34 (n = 131)	0.16 ± 0.99 (p = 0.07)	3.31 ± 1.71 (n = 130)	0.19 ± 1.16 (p = 0.07)	0.10	− 0.02 (− 0.29, 0.24) (p = 0.86)
eGFR (mL/min/1.73m^2^)	Week 0	80.6 ± 20.7 (n = 145)		81.6 ± 24.6 (n = 144)		0.69	
	Week 104	77.6 ± 19.4 (n = 146)	− 2.9 ± 11.9 (p = 0.004)	78.2 ± 23.2 (n = 145)	− 3.4 ± 10.3 (p < 0.001)	0.82	0.4 (− 2.1, 3.0) (p = 0.74)
	Week 208	74.4 ± 18.2 (n = 143)	− 6.4 ± 12.7 (p < 0.001)	73.9 ± 22.7 (n = 137)	− 8.4 ± 13.3 (p < 0.001)	0.84	1.9 (− 1.2, 5.0) (p = 0.22)
Total cholesterol (mmol/L)	Week 0	4.97 ± 0.74 (n = 144)		4.92 ± 0.78 (n = 140)		0.57	
	Week 104	5.04 ± 0.87 (n = 145)	0.07 ± 0.68 (p = 0.20)	4.86 ± 0.85 (n = 139)	− 0.05 ± 0.66 (p = 0.33)	0.08	0.13 (− 0.03, 0.28) (p = 0.11)
	Week 208	4.96 ± 0.94 (n = 141)	0.01 ± 0.79 (p = 0.87)	4.74 ± 0.91 (n = 136)	− 0.17 ± 0.81 (p = 0.020)	0.045	0.18 (− 0.01, 0.37) (p = 0.07)
LDL-C (mmol/L)	Week 0	2.90 ± 0.69 (n = 145)		2.87 ± 0.62 (n = 145)		0.71	
	Week 104	2.91 ± 0.77 (n = 144)	0.00 ± 0.63 (p = 0.96)	2.81 ± 0.70 (n = 144)	− 0.07 ± 0.55 (p = 0.14)	0.26	0.07 (− 0.07, 0.20) (p = 0.35)
	Week 208	2.79 ± 0.76 (n = 140)	− 0.11 ± 0.75 (p = 0.07)	2.70 ± 0.76 (n = 136)	− 0.16 ± 0.73 (p = 0.014)	0.35	0.04 (− 0.13, 0.22) (p = 0.64)
HDL-C (mmol/L)	Week 0	1.43 ± 0.36 (n = 146)		1.38 ± 0.31 (n = 145)		0.23	
	Week 104	1.50 ± 0.36 (n = 146)	0.07 ± 0.20 (p < 0.001)	1.42 ± 0.34 (n = 145)	0.04 ± 0.20 (p = 0.016)	0.06	0.03 (− 0.02, 0.08) (p = 0.20)
	Week 208	1.52 ± 0.39 (n = 144)	0.09 ± 0.21 (p < 0.001)	1.41 ± 0.34 (n = 140)	0.04 ± 0.23 (p = 0.037)	0.018	0.05 (0.00, 0.11) (p = 0.039)
Triglyceride (ln(mmol/L))	Week 0	0.24 ± 0.51 (n = 146)		0.34 ± 0.44 (n = 145)		0.09	
	Week 104	0.21 ± 0.51 (n = 139)	− 0.03 ± 0.38 (p = 0.42)	0.28 ± 0.48 (n = 140)	− 0.04 ± 0.41 (p = 0.20)	0.24	0.02 (− 0.07, 0.11) (p = 0.69)
	Week 208	0.19 ± 0.50 (n = 129)	− 0.02 ± 0.41 (p = 0.52)	0.27 ± 0.51 (n = 126)	− 0.06 ± 0.40 (p = 0.08)	0.24	0.04 (− 0.06, 0.14) (p = 0.44)
Systolic blood pressure (mmHg)	Week 0	133.5 ± 14.6 (n = 142)		134.1 ± 15.7 (n = 140)		0.74	
	Week 104	128.5 ± 14.5 (n = 145)	− 4.9 ± 15.8 (p < 0.001)	134.3 ± 17.5 (n = 144)	0.4 ± 17.3 (p = 0.80)	0.002	− 5.3 (− 9.2, − 1.4) (p = 0.008)
	Week 208	127.9 ± 15.4 (n = 143)	− 6.0 ± 15.7 (p < 0.001)	132.7 ± 16.9 (n = 137)	− 1.1 ± 19.6 (p = 0.52)	0.013	− 4.9 (− 9.1, − 0.7) (p = 0.023)
Diastolic blood pressure (mmHg)	Week 0	78.1 ± 10.3 (n = 142)		79.1 ± 10.5 (n = 140)		0.45	
	Week 104	74.9 ± 10.4 (n = 145)	− 3.3 ± 9.9 (p < 0.001)	77.8 ± 9.9 (n = 144)	− 1.3 ± 9.6 (p = 0.13)	0.015	− 2.0 (− 4.3, 0.3) (p = 0.08)
	Week 208	74.8 ± 11.7 (n = 143)	− 3.2 ± 12.2 (p = 0.002)	77.4 ± 10.7 (n = 137)	− 1.9 ± 12.2 (p = 0.08)	0.05	− 1.4 (− 4.3, 1.5) (p = 0.35)

Data are presented as the mean ± standard deviation. Intragroup differences from the baseline were analyzed using a one-sample t-test. Intergroup differences in changes from baseline (treatment effect) were analyzed using Student’s t-test

HbA1c: hemoglobin A1c; UAE: urinary albumin excretion; eGFR: estimated glomerular filtration rate; LDL-C: low-density lipoprotein cholesterol; HDL-C: high-density lipoprotein cholesterol; 95% CI: 95% confidence interval

### Renal function and lipid metabolism

Serum creatinine levels significantly increased and the eGFR significantly decreased in both groups throughout the follow-up period, but no significant intergroup differences in the changes were observed (Table 3).

Total cholesterol and LDL-C levels significantly decreased in the conventional treatment group but not in the tofogliflozin group throughout the follow-up period, although no significant intergroup differences in the changes were observed (Table 3). HDL-C levels significantly increased in both groups throughout the main UTOPIA study and the follow-up period, and a significant intergroup difference in changes was observed at week 208; the increase was larger in the tofogliflozin group than in the conventional treatment group. Triglyceride levels did not change significantly in either group throughout the follow-up period.

### BMI and blood pressure

The BMI was significantly decreased in the tofogliflozin group throughout the follow-up period but did not change significantly during the main UTOPIA study. However, in the conventional treatment group, the BMI decreased significantly from baseline to the end of the UTOPIA Extension study, resulting in a significant intergroup difference in changes throughout the follow-up period (Table 3). Waist circumference was significantly decreased in the tofogliflozin group throughout the follow-up period but was unchanged in the conventional treatment group. There was a significant intergroup difference in changes throughout the follow-up period.

Systolic and diastolic blood pressure were significantly decreased throughout the follow-up period in the tofogliflozin group, but no significant intragroup change in blood pressure was observed throughout the follow-up period in the conventional treatment group. Although a significant intergroup difference in the changes in systolic blood pressure was observed throughout the follow-up period, no significant intergroup difference in the changes in diastolic blood pressure was observed throughout the follow-up period.

### Safety

The frequency of cardiovascular events was compared between the groups. The frequency of the predefined composite endpoint comprising cardiovascular mortality, acute myocardial infarction, unstable angina, and heart failure was significantly lower in the tofogliflozin group than in the conventional treatment group in the log-rank test (Additional file 4. The frequency of study agent-related events (hypoglycemia, urinary tract and genital infection, skin manifestations, and body fluid reduction) (Additional file 5) and renal events (Additional file 6) did not significantly differ between the groups.

The adverse events (AEs) that occurred during the study are listed in Additional file 7. During the follow-up period, 88 participants (60.3%) in the tofogliflozin group and 96 participants (66.2%) in the conventional treatment group developed AEs. Furthermore, 45 participants (30.8%) in the tofogliflozin group and 47 participants (32.4%) in the conventional treatment group developed serious AEs. The overall incidence of AEs and serious AEs as well as the incidence of each AE did not vary significantly between the groups.

## Discussion

This UTOPIA Extension study showed that tofogliflozin was not associated with improved inhibition of carotid wall thickening but induced long-term attenuation of baPWV progression and improvement of HbA1c and HDL-C levels, BMI, waist circumference, and systolic blood pressure while maintaining a good safety profile. This study is the first to demonstrate that the beneficial effect of SGLT2 inhibitors on arterial stiffness lasts for a long time (more than 4 years).

The main UTOPIA study was the first RCT to assess the effect of SGLT2 inhibitors on carotid atherosclerosis, which demonstrated a significant decrease in IMT with tofogliflozin treatment. Similarly, in this UTOPIA Extension study, a significant reduction in the mean IMT-CCA and a tendency towards decreased right and left max-IMT-CCA were maintained throughout the follow-up period in the tofogliflozin treatment group (Table 2). However, as a significant IMT reduction was also observed in the control group, no significant intergroup difference was detected in both the main and extended UTOPIA studies. Thus, the series of UTOPIA studies have not demonstrated a beneficial effect of tofogliflozin on carotid atherosclerosis.

One possible explanation for these findings is that the effect of tofogliflozin on the atherosclerotic changes that occur in the large arteries, including the carotid arteries, is limited. Indeed, a sub-analysis of the main UTOPIA trial has recently shown that the tissue characteristics of the carotid arterial wall did not change in either the tofogliflozin or conventional treatment groups during a 104-week treatment period [24]. In contrast, Irace et al. reported a significant decrease in carotid IMT after only 3 months of treatment with empagliflozin, another SGLT2 inhibitor [25]. They suggested that this early reduction in IMT may be related to hemodynamic changes that occur during empagliflozin treatment. These findings may be consistent with the fact that although RCTs of SGLT2 inhibitors reported decreased cardiovascular events [6, 7], these treatments did not reduce atherothrombotic events such as myocardial infarction and stroke [6, 7, 26].

It is also possible that the beneficial effects of tofogliflozin on the carotid wall, if any, were masked by the administration of additional antidiabetic, antihypertensive, and antilipidemic agents. Biguanides [27, 28], dipeptidyl peptidase-4 inhibitors [22, 23], angiotensin II receptor blockers [29, 30], and antilipidemic agents [31, 32] were reported to have inhibitory effects on the progression of IMT thickening, and more than half the participants in the UTOPIA Extension study used these drugs. Moreover, there was a difference between the two groups in the rates of biguanide and dipeptidyl peptidase-4 inhibitor use during the observation period, although the difference was not statistically significant. Similarly, there were imbalances in the administration of angiotensin II receptor blockers (37.7% and 50.3% in the tofogliflozin and conventional treatment groups at baseline (p = 0.034), respectively) and lipid-lowering agents (49.3% and 60.0% in the tofogliflozin and conventional treatment groups at baseline (p = 0.08), respectively), and these imbalances were observed throughout the study period. Further trials are required to evaluate the effect of SGLT2 inhibitors on IMT progression in first-line therapy with SGLT2 inhibitors.

In contrast, the increase in baPWV, an indicator of arterial stiffness, was significantly attenuated with tofogliflozin treatment but not with conventional treatment in both the UTOPIA main and extension studies (Table 2). baPWV is associated with various cardiovascular risk factors [33, 34] and could be a predictor of future cardiovascular events. Evidence on the effect of SGLT2 inhibitors on arterial stiffness remains controversial. Some studies reported beneficial effects of other SGLT2 inhibitors similar to those observed in the UTOPIA main and extension studies using other indices of arterial stiffness [35–37], whereas others reported no benefit of SGLT2 inhibitors on arterial stiffness [38]. However, the observation periods in these previous studies were relatively short.

The results of the UTOPIA main and extension studies indicate that tofogliflozin could have different effects on IMT and baPWV. IMT and baPWV are both indicators of atherosclerosis; however, they are believed to reflect different aspects of cardiovascular risk. Carotid IMT primarily reflects the degree of atherosclerosis, particularly structural changes that develop in large arteries [39]. In contrast, baPWV reflects deterioration in the elasticity of arterial walls due to functional and structural arteriosclerotic changes that occur in the area extending from the aorta to the arteries of the extremities [40, 41]. Loss of arterial wall elasticity increases vascular wall stress, resulting in atherosclerosis, elevated left ventricular afterload, left ventricular diastolic dysfunction, and coronary perfusion disorder. Therefore, baPWV may reflect not only atherosclerotic changes but also the risk for various cardiovascular disorders, including cardiac dysfunction.

Recently, there has been a growing number of research reports suggesting a cardiovascular protective effect of tofogliflozin. Kishima et al. conducted a prospective RCT to compare the suppressive effects of tofogliflozin and anagliptin, a DPP-4 inhibitor, on atrial fibrillation recurrence after catheter ablation in patients with type 2 diabetes mellitus. In their study, greater suppression of atrial fibrillation recurrence was achieved with tofogliflozin than with anagliptin [42]. In addition, Joki et al. reported that tofogliflozin treatment ameliorated right heart overload and pulmonary vascular remodeling in an experimental rodent model [43]. To compare subsequent cardiovascular risks, including heart failure, myocardial infarction, angina pectoris, stroke, and atrial fibrillation, following the use of different SGLT2 inhibitors, Suzuki et al. analyzed a large-scale real-world dataset comprising approximately 25,000 patients with diabetes mellitus who were newly prescribed SGLT2 inhibitors (empagliflozin, dapagliflozin, canagliflozin, ipragliflozin, tofogliflozin, and luseogliflozin) in Japan. The risk for subsequent development of these outcomes was comparable between the individual SGLT2 inhibitors [44]. Despite the lack of RCTs on tofogliflozin with atherosclerotic cardiovascular disease or heart failure as primary outcomes and, thus, the lack of sufficient evidence, it is likely that tofogliflozin, like other SGLT2 inhibitors, has a beneficial effect on cardiovascular disease, particularly on cardiac function and heart failure.

In this study, the tofogliflozin group showed significantly lower HbA1c levels at week 208 compared with baseline, and this reduction in HbA1c levels was significantly greater than that in the conventional treatment group. Throughout the observation period, there was no difference between the treatment groups in the proportion of patients concomitantly receiving other diabetes medications. This suggests that the effect of tofogliflozin on glycemic control lasts at least 4 years. Although tofogliflozin did not affect serum TC, LDL-C, and TG levels, it elevated serum HDL-C levels compared to conventional treatment, even during the UTOPIA Extension study (Table 3). As low HDL-C levels are considered a cardiovascular risk factor, the long-lasting effect of tofogliflozin on serum HDL-C levels, which persisted for years, may lead to cardiovascular protection. Tofogliflozin treatment was also associated with a significant reduction in body weight, waist circumference, and systolic and diastolic blood pressure, consistent with previous reports indicating the favorable hemodynamic effects of SGLT2 inhibitors.

Tofogliflozin did not affect renal function biomarkers (serum creatinine, UAE, and eGFR) in this study. This was not consistent with previous reports demonstrating the renal benefits of SGLT2 inhibitors [5, 7, 45]. In a previous study, the renal benefits of SGLT2 inhibitors were more prominent in patients with a lower eGFR at baseline than in others [46]; hence, one reason for this inconsistency might be the relatively normal to mildly-impaired renal function of patients in this study: the baseline eGFR was 80.6 ± 20.7 mL/min/1.73 m^2^ and 81.6 ± 24.6 mL/min/1.73 m^2^ in the tofogliflozin and conventional treatment groups, respectively.

Regarding safety, during the 2-year intervention with tofogliflozin and 2-year extensional observation, the frequency of all adverse events did not vary between the groups. However, the frequency of the composite cardiovascular endpoint comprising cardiovascular mortality, acute myocardial infarction, unstable angina, and heart failure was significantly lower in the tofogliflozin group than in the conventional treatment group (Additional file 4), suggesting a long-term effect of tofogliflozin on the suppression of cardiovascular events.

Collectively, these results indicate that tofogliflozin treatment improves various cardiovascular risk factors and that such beneficial effects persist for at least 4 years, suggesting that tofogliflozin should be used early in diabetes management.

This study had several limitations. First, the main UTOPIA study was not a double-blind, placebo-controlled trial but rather a prospective, open-label, randomized trial with a blinded endpoint. In addition, the UTOPIA Extension study is a prospective observational study without any restriction in medication in both groups. Although the assessment of endpoints was blinded and conducted by expert committees, the study design may have caused unexpected bias. Second, all participants in this study were Japanese. Therefore, generalizing the results of this study to other racial or ethnic groups should be approached with caution. Finally, we did not correct for multiplicity in this study. This may lead to false-positive results.

## Conclusions

This is the first study to demonstrate that tofogliflozin is not associated with improved inhibition of carotid wall thickening but exerts long-term positive effects on various cardiovascular risk factors including HbA1c and HDL-C levels, BMI, waist circumference, systolic blood pressure, and baPWV while showing a good safety profile.

## Supplementary Information

**Additional file 1. **List of study investigators.

**Additional file 2. **Effects of tofogliflozin on ABI.

**Additional file 3. **Changes in concomitantly used glucose-lowering agent.

**Additional file 4. **Frequency of cardiovascular events.

**Additional file 5. **Frequency of study agent-related events.

**Additional file 6. **Frequency of renal events.

**Additional file 7. **Adverse events.

## Data Availability

The datasets generated and/or analyzed during our study are available from the corresponding author upon reasonable request.

## Abbreviations

ABI: Ankle-brachial index
AE: Adverse events
ANCOVA: Analysis of covariance
baPWV: Brachial-ankle pulse wave velocity
BMI: Body mass index
eGFR: Estimated glomerular filtration rate
HbA1c: Hemoglobin A1c
HDL-C: High-density lipoprotein cholesterol
IMT: Intima-media thickness
LDL-C: Low-density lipoprotein cholesterol
SD: Standard deviation
SGLT2: Sodium-glucose cotransporter 2
TG: Triglyceride
UAE: Urinary albumin excretion
