# Supplementary material for: Tofogliflozin long-term effects on atherosclerosis progression and major clinical parameters in patients with type 2 diabetes mellitus lacking a history of cardiovascular disease: a 2-year extension study of the UTOPIA trial
Source: Cardiovasc Diabetol. 2023 Jun 22;22:143. doi: 10.1186/s12933-023-01879-4 (PMC10286339; doi:10.1186/s12933-023-01879-4)
Supplement: Supplementary file 1 — Additional file 1. List of study investigators. [file 12933_2023_1879_MOESM1_ESM.docx]

**Additional file 1**

UTOPIA Extension study site investigators (listed in alphabetical order)

Hayashi Clinic: I Hayashi

Ikeda Municipal Hospital: M Tsugawa

Jiyugaoka Medical Clinic: H Yokoyama

Juntendo Tokyo Koto Geriatric Medical Center: H Yoshii

Juntendo University Graduate School of Medicine: K Komiyama, T Mita, T Shimizu

Kansai Rosai Hospital: T Yamamoto

Kanda Naika Clinic: S Kawashima

Kawasaki Hospital: T Nakamura

Kawasaki Medical School: S Kamei, T Kinoshita, M Shimoda

Kitasenri Maeda Clinic: K Maeda

Kosugi Medical Clinic: K Kosugi

Misaki Naika Clinic: H Yoshii

Nakakinen Clinic: T Osonoi, M Saito

Nippon Life Hospital: S Sumitani

Osaka General Medical Center: N Fujiki, Y Fujita, S Shimizu, Y Umayahara, M Hatazaki

National Hospital Organization Osaka National Hospital: K Kato

Osaka Police Hospital: Y Irie, R Kataoka, T Yasuda

Osaka Rosai Hospital: Y Kiyohara, M Ohashi, K Ryomoto, Y Takahi

Osaka University Graduate School of Medicine: Y Fujishima, Y Fujita, A Fukuhara, K Fukui, Y Hosokawa, A Imagawa, H Iwahashi, K Mukai, N Katakami, T Katsura, D Kawamori, T Kimura, S Kobayashi, J Kozawa, F Kubo, N Maeda, T Matsuoka, K Miyashita, S Nakata, H Ninomiya, H Nishizawa, Y Okuno, M Otsuki, F Sakamoto, S Sasaki, I Sato, N Shimo, I Shimomura, M Takahara, T Takano, A Tokunaga, S Uno, M Yamaoka, S Yoneda

Otoshi Medical Clinic: K Ohtoshi

Shiraiwa Medical Clinic: T Shiraiwa

University of Occupational and Environmental Health, Japan: M Hajime, K Koikawa, F Kuno, A Kurozumi, K Matsushita, M Narisawa, K Tanaka, K Sugai, Y Okada, K Torimoto, A Kurozumi
