# Supplementary material for: Tofogliflozin long-term effects on atherosclerosis progression and major clinical parameters in patients with type 2 diabetes mellitus lacking a history of cardiovascular disease: a 2-year extension study of the UTOPIA trial
Source: Cardiovasc Diabetol. 2023 Jun 22;22:143. doi: 10.1186/s12933-023-01879-4 (PMC10286339; doi:10.1186/s12933-023-01879-4)
Supplement: Supplementary file 2 — Additional file 2. Effects of tofogliflozin on ABI. [file 12933_2023_1879_MOESM2_ESM.docx]

**Additional file 2.** Effects of tofogliflozin on ABI

|  | Observation point | Level | Tofogliflozin group | | Conventional treatment group | | Intergroup p value | |
| --- | --- | --- | --- | --- | --- | --- | --- | --- |
|  |  |  | N | n (%) | N | n (%) | Chi-square | Fisher |
| ABI right | Week 0 | Abnormally low (ABI≤0.9) | 104 | 2 (1.9) | 95 | 1 (1.1) | 0.88* | 1.00 |
|  |  | Within normal range (0.9<ABI<1.3) |  | 99 (95.2) |  | 91 (95.8) |  |  |
|  |  | Abnormally high (1.3≤ABI) |  | 3 (2.9) |  | 3 (3.2) |  |  |
|  | Week 104 | Abnormally low (ABI≤0.9) | 97 | 1 (1.0) | 88 | 0 (0.0) | 0.56* | 0.85 |
|  |  | Within normal range (0.9<ABI<1.3) |  | 93 (95.9) |  | 84 (95.5) |  |  |
|  |  | Abnormally high (1.3≤ABI) |  | 3 (3.1) |  | 4 (4.5) |  |  |
|  | Week 208 | Abnormally low (ABI≤0.9) | 88 | 0 (0.0) | 73 | 1 (1.4) | 0.52* | 0.73 |
|  |  | Within normal range (0.9<ABI<1.3) |  | 82 (93.2) |  | 68 (93.2) |  |  |
|  |  | Abnormally high (1.3≤ABI) |  | 6 (6.8) |  | 4 (5.5) |  |  |
| ABI left | Week 0 | Abnormally low (ABI≤0.9) | 103 | 3 (2.9) | 95 | 1 (1.1) | 0.53* | 0.61 |
|  |  | Within normal range (0.9<ABI<1.3) |  | 99 (96.1) |  | 92 (96.8) |  |  |
|  |  | Abnormally high (1.3≤ABI) |  | 1 (1.0) |  | 2 (2.1) |  |  |
|  | Week 104 | Abnormally low (ABI≤0.9) | 97 | 1 (1.0) | 88 | 2 (2.3) | 0.57* | 0.57 |
|  |  | Within normal range (0.9<ABI<1.3) |  | 92 (94.8) |  | 80 (90.9) |  |  |
|  |  | Abnormally high (1.3≤ABI) |  | 4 (4.1) |  | 6 (6.8) |  |  |
|  | Week 208 | Abnormally low (ABI≤0.9) | 87 | 3 (3.4) | 73 | 0 (0.0) | 0.27* | 0.39 |
|  |  | Within normal range (0.9<ABI<1.3) |  | 79 (90.8) |  | 68 (93.2) |  |  |
|  |  | Abnormally high (1.3≤ABI) |  | 5 (5.7) |  | 5 (6.8) |  |  |

Data are presented as number (%) of patients.

Chi-square test or Fisher’s exact test were performed for intergroup comparisons.

*Did not meet the requirement for the chi-square test.

ABI, ankle-brachial index
