# Supplementary material for: Tofogliflozin long-term effects on atherosclerosis progression and major clinical parameters in patients with type 2 diabetes mellitus lacking a history of cardiovascular disease: a 2-year extension study of the UTOPIA trial
Source: Cardiovasc Diabetol. 2023 Jun 22;22:143. doi: 10.1186/s12933-023-01879-4 (PMC10286339; doi:10.1186/s12933-023-01879-4)
Supplement: Supplementary file 3 — Additional file 3. Changes in concomitantly used glucose-lowering agent. [file 12933_2023_1879_MOESM3_ESM.docx]

**Additional file 3.** Changes in concomitantly used glucose-lowering agent

| Parameters | Tofogliflozin treatment group  (n=146) | Conventional treatment group  (n=145) | p value |
| --- | --- | --- | --- |
| Any concomitantly used glucose-lowering agent* |  |  |  |
| Baseline | 133 (91.1) | 129 (89.0) | 0.56 |
| Week 26 | 132 (90.4) | 132 (91.0) | 1.00 |
| Week 52 | 131 (89.7) | 132 (91.0) | 0.84 |
| Week 78 | 131 (89.7) | 133 (91.7) | 0.69 |
| Week 104 | 130 (89.0) | 133 (91.7) | 0.55 |
| Week 156 | 132 (90.4) | 134 (93.7) | 0.39 |
| Week 208 | 133 (91.1) | 131 (92.9) | 0.67 |
| Metformin |  |  |  |
| Baseline | 78 (53.4) | 88 (60.7) | 0.24 |
| Week 26 | 78 (53.4) | 93 (64.1) | 0.07 |
| Week 52 | 77 (52.7) | 97 (66.9) | 0.017 |
| Week 78 | 78 (53.4) | 96 (66.2) | 0.031 |
| Week 104 | 78 (53.4) | 97 (66.9) | 0.023 |
| Week 156 | 85 (58.2) | 102 (71.3) | 0.026 |
| Week 208 | 88 (60.3) | 98 (69.5) | 0.11 |
| Sulfonylurea |  |  |  |
| Baseline | 35 (24.0) | 39 (26.9) | 0.59 |
| Week 26 | 31 (21.2) | 38 (26.2) | 0.34 |
| Week 52 | 32 (21·9) | 43 (29.7) | 0.14 |
| Week 78 | 32 (21·9) | 41 (28.3) | 0.23 |
| Week 104 | 30 (20.5) | 41 (28.3) | 0.13 |
| Week 156 | 30 (20.5) | 35 (24.5) | 0.48 |
| Week 208 | 34 (23.3) | 30 (21.3) | 0.78 |
| Glinides |  |  |  |
| Baseline | 8 (5.5) | 9 (6.2) | 0.81 |
| Week 26 | 7 (4.8) | 10 (6.9) | 0.47 |
| Week 52 | 7 (4.8) | 8 (5.5) | 0.80 |
| Week 78 | 7 (4.8) | 8 (5.5) | 0.80 |
| Week 104 | 8 (5.5) | 8 (5.5) | 1.00 |
| Week 156 | 8 (5.5) | 9 (6.3) | 0.81 |
| Week 208 | 8 (5.5) | 10 (7.1) | 0.63 |
| Thiazolidinediones |  |  |  |
| Baseline | 17 (11.6) | 19 (13.1) | 0.73 |
| Week 26 | 16 (11.0) | 20 (13.8) | 0.48 |
| Week 52 | 17 (11.6) | 21 (14.5) | 0.49 |
| Week 78 | 17 (11.6) | 21 (14.5) | 0.49 |
| Week 104 | 17 (11.6) | 20 (13.8) | 0.60 |
| Week 156 | 17 (11.6) | 20 (14.0) | 0.60 |
| Week 208 | 17 (11.6) | 16 (11.3) | 1.00 |
| α-glucosidase inhibitors |  |  |  |
| Baseline | 22 (15.1) | 21 (14.5) | 1.00 |
| Week 26 | 23 (15.8) | 23 (15.9) | 1.00 |
| Week 52 | 23 (15.8) | 21 (14.5) | 0.87 |
| Week 78 | 23 (15.8) | 22 (15.2) | 1.00 |
| Week 104 | 22 (15.1) | 23 (15.9) | 0.87 |
| Week 156 | 21 (14.8) | 22 (15.4) | 0.87 |
| Week 208 | 23 (15.8) | 21 (14.9) | 0.87 |
| DPP-4 inhibitors |  |  |  |
| Baseline | 68 (46.6) | 82 (56.6) | 0.10 |
| Week 26 | 67 (45.9) | 85 (58.6) | 0.035 |
| Week 52 | 68 (46.6) | 83 (57.2) | 0.08 |
| Week 78 | 68 (46.6) | 82 (56.6) | 0.08 |
| Week 104 | 66 (45.2) | 82 (56.6) | 0.06 |
| Week 156 | 73 (50.0) | 85 (59.4) | 0.12 |
| Week 208 | 72 (49.3) | 82 (58.2) | 0.16 |
| GLP-1 R agonists |  |  |  |
| Baseline | 19 (13.0) | 10 (6.9) | 0.12 |
| Week 26 | 19 (13.0) | 12 (8.3) | 0.25 |
| Week 52 | 19 (13.0) | 13 (9.0) | 0.35 |
| Week 78 | 19 (13.0) | 12 (8.3) | 0.25 |
| Week 104 | 19 (13.0) | 12 (8.3) | 0.25 |
| Week 156 | 22 (15.1) | 14 (9.7) | 0.21 |
| Week 208 | 25 (17.1) | 16 (11.3) | 0.18 |
| Insulins |  |  |  |
| Baseline | 27 (18.5) | 32 (22.1) | 0.47 |
| Week 26 | 28 (19.2) | 32 (22.1) | 0.56 |
| Week 52 | 28 (19.2) | 33 (22.8) | 0.47 |
| Week 78 | 28 (19.2) | 32 (22.1) | 0.56 |
| Week 104 | 28 (19.2) | 32 (22.1) | 0.56 |
| Week 156 | 25 (17.1) | 33 (22.9) | 0.24 |
| Week 208 | 28 (19.2) | 37 (26.2) | 0.16 |

Data are presented as number (%) of patients. The two treatment groups were compared by Fisher’s exact test.

* Tofogliflozin was not counted among concomitantly used anti-diabetic agents in the tofogliflozin treatment group.
