# Supplementary material for: Tofogliflozin long-term effects on atherosclerosis progression and major clinical parameters in patients with type 2 diabetes mellitus lacking a history of cardiovascular disease: a 2-year extension study of the UTOPIA trial
Source: Cardiovasc Diabetol. 2023 Jun 22;22:143. doi: 10.1186/s12933-023-01879-4 (PMC10286339; doi:10.1186/s12933-023-01879-4)
Supplement: Supplementary file 4 — Additional file 4. Frequency of cardiovascular events. [file 12933_2023_1879_MOESM4_ESM.docx]

**Additional file 4.** Frequency of cardiovascular events

| Cardiovascular events | | Tofogliflozin group  (n=145) | Conventional treatment group  (n=145) | log-rank p value | HR (95% CI) |
| --- | --- | --- | --- | --- | --- |
| a | Ischemic heart disease  (sudden cardiac death,  acute myocardial infarction,  unstable angina, and  execution of coronary revascularization) | 4 (2.8) | 5 (3.4) | 0.74 | 0.80 (0.22, 2.99) |
| b | Cerebrovascular disorder  (cerebral infarction,  intracerebral hemorrhage, and  subarachnoid hemorrhage) | 2 (1.4) | 2 (1.4) | 0.98 | 0.98 (0.14, 6.95) |
| c | Arterial disease  (arteriosclerosis obliterans and  leg amputation) | 3 (2.1) | 0 (0.0) | 0.09 | 29247003.59 (0.00, -) |
| d | Heart failure | 0 (0.0) | 1 (0.7) | 0.32 | 0.00 (0.00, -) |
| e | a + b + c + d | 7 (4.8) | 7 (4.8) | 0.98 | 0.99 (0.35, 2.81) |
| f | a + b + c + all-cause mortality | 7 (4.8) | 8 (5.5) | 0.78 | 0.86 (0.31, 2.38) |
| g | a + b + c + d + all-cause mortality | 7 (4.8) | 8 (5.5) | 0.78 | 0.86 (0.31, 2.38) |
| h | Cardiovascular mortality +  acute myocardial infarction +  cerebral infarction | 0 (0.0) | 2 (1.4) | 0.15 | 0.00 (0.00, -) |
| i | Cardiovascular mortality +  acute myocardial infarction +  unstable angina +  cerebral infarction | 0 (0.0) | 3 (2.1) | 0.08 | 0.00 (0.00, -) |
| j | Cardiovascular mortality +  acute myocardial infarction +  cerebral infarction +  heart failure | 0 (0.0) | 3 (2.1) | 0.08 | 0.00 (0.00, -) |
| k | Cardiovascular mortality +  acute myocardial infarction +  unstable angina +  heart failure | 0 (0.0) | 4 (2.8) | 0.043 | 0.00 (0.00, -) |

Data are presented as number (%) of patients.

95% CI, 95% confidence interval; HR, hazard ratio
