# Supplementary material for: Tofogliflozin long-term effects on atherosclerosis progression and major clinical parameters in patients with type 2 diabetes mellitus lacking a history of cardiovascular disease: a 2-year extension study of the UTOPIA trial
Source: Cardiovasc Diabetol. 2023 Jun 22;22:143. doi: 10.1186/s12933-023-01879-4 (PMC10286339; doi:10.1186/s12933-023-01879-4)
Supplement: Supplementary file 5 — Additional file 5. Frequency of study agent-related events. [file 12933_2023_1879_MOESM5_ESM.docx]

**Additional file 5.** Frequency of study agent-related events

| Event | | Tofogliflozin group | | Conventional treatment group | | log-rank p value | HR (95% CI) |
| --- | --- | --- | --- | --- | --- | --- | --- |
|  |  | n | Frequency | n | Frequency |  |  |
| a | Hypoglycemia | 144 | 17 (11.8) | 143 | 17 (11.9) | 0.99 | 1.01 (0.51, 1.97) |
| b | Urinary tract infection, genital infection | 145 | 9 (6.2) | 145 | 8 (5.5) | 0.81 | 1.13 (0.43, 2.92) |
| c | Skin manifestation | 145 | 10 (6.9) | 145 | 10 (6.9) | 1.00 | 1.00 (0.42, 2.40) |
| d | Body fluid reduction-related event  (polyuria,  frequent urination, and  dehydration) | 145 | 3 (2.1) | 145 | 0 (0.0) | 0.08 | 29587149.15 (0.00, -) |

Data are presented as number (%) of patients.

95% CI, 95% confidence interval; HR, hazard ratio
