# Supplementary material for: Tofogliflozin long-term effects on atherosclerosis progression and major clinical parameters in patients with type 2 diabetes mellitus lacking a history of cardiovascular disease: a 2-year extension study of the UTOPIA trial
Source: Cardiovasc Diabetol. 2023 Jun 22;22:143. doi: 10.1186/s12933-023-01879-4 (PMC10286339; doi:10.1186/s12933-023-01879-4)
Supplement: Supplementary file 6 — Additional file 6. Frequency of renal events. [file 12933_2023_1879_MOESM6_ESM.docx]

**Additional file 6.** Frequency of renal events

| Event | | Tofogliflozin group | | Conventional treatment group | | log-rank p value | HR (95% CI) |
| --- | --- | --- | --- | --- | --- | --- | --- |
|  |  | n | Frequency | n | Frequency |  |  |
| a | Decrease of eGFR 30% or higher from baseline | 144 | 8 (5.6) | 144 | 12 (8.3) | 0.34 | 0.65 (0.26, 1.58) |
| b | Increase of serum creatinine twice or higher from baseline | 144 | 0 (0.0) | 144 | 2 (1.4) | 0.15 | 0.00 (0.00, -) |
| c | Improvement of disease stage category of nephropathy based on UAE from baseline | 137 | 25 (18.2) | 134 | 19 (14.2) | 0.35 | 1.33 (0.73, 2.41) |
| d | Worsening of disease stage category of nephropathy based on UAE from baseline | 137 | 35 (25.5) | 134 | 40 (29.9) | 0.38 | 0.82 (0.52, 1.28) |
| e | End-stage renal disease  (dialysis,  renal transplantation, or  eGFR < 15 mL/min/1.73m^2^) | 145 | 0 (0.0) | 145 | 1 (0.7) | 0.31 | 0.00 (0.00, -) |
| f | Death caused by renal dysfunction or cardiovascular death | 145 | 0 (0.0) | 145 | 0 (0.0) | - | - |
| g | Renal composite event (a + d) | 137 | 39 (28.5) | 134 | 48 (35.8) | 0.16 | 0.74 (0.49, 1.13) |
| h | Renal composite event (a + e + f) | 144 | 8 (5.6) | 144 | 12 (8.3) | 0.34 | 0.65 (0.26, 1.58) |

Data are presented as number (%) of patients.

UAE, urinary albumin excretion; eGFR, estimated glomerular filtration rate; 95% CI, 95% confidence interval; HR, hazard ratio
