# Supplementary material for: Tofogliflozin long-term effects on atherosclerosis progression and major clinical parameters in patients with type 2 diabetes mellitus lacking a history of cardiovascular disease: a 2-year extension study of the UTOPIA trial
Source: Cardiovasc Diabetol. 2023 Jun 22;22:143. doi: 10.1186/s12933-023-01879-4 (PMC10286339; doi:10.1186/s12933-023-01879-4)
Supplement: Supplementary file 7 — Additional file 7. Adverse events. [file 12933_2023_1879_MOESM7_ESM.docx]

# Additional file 7. Adverse events

|  | Tofogliflozin group | | Conventional treatment group | |
| --- | --- | --- | --- | --- |
| Number of subjects in the safety analysis set | 146 | | 145 | |
| Death | 0 (0.0) | | 1 (0.7) | |
| Any adverse event | 88 (60.3) | | 96 (66.2) | |
| Any serious adverse event | 45 (30.8) | | 47 (32.4) | |
|  | Total | Serious | Total | Serious |
| Worsening of glycemic control | 20 (13.7) | 7 (4.8) | 32 (22.1) | 10 (6.9) |
| Common cold | 19 (13.0) | 0 (0.0) | 19 (13.1) | 0 (0.0) |
| Hypoglycemia | 18 (12.3) | 0 (0.0) | 19 (13.1) | 0 (0.0) |
| Bone fracture | 12 (8.2) | 5 (3.4) | 5 (3.4) | 3 (2.1) |
| Cataract | 6 (4.1) | 2 (1.4) | 6 (4.1) | 4 (2.8) |
| Constipation | 5 (3.4) | 0 (0.0) | 7 (4.8) | 0 (0.0) |
| Gastroesophageal reflux disease | 5 (3.4) | 0 (0.0) | 1 (0.7) | 0 (0.0) |
| Colonic polyp | 4 (2.7) | 2 (1.4) | 5 (3.4) | 4 (2.8) |
| Influenza | 4 (2.7) | 1 (0.7) | 5 (3.4) | 0 (0.0) |
| Sinusitis | 3 (2.1) | 1 (0.7) | 0 (0.0) | 0 (0.0) |
| Cystitis | 3 (2.1) | 0 (0.0) | 4 (2.8) | 0 (0.0) |
| Gastritis | 3 (2.1) | 0 (0.0) | 2 (1.4) | 0 (0.0) |
| Allergic rhinitis | 3 (2.1) | 0 (0.0) | 1 (0.7) | 0 (0.0) |
| Epigastric discomfort | 3 (2.1) | 0 (0.0) | 1 (0.7) | 0 (0.0) |
| Vulvovaginal candidiasis | 3 (2.1) | 0 (0.0) | 0 (0.0) | 0 (0.0) |
| Obesity | 2 (1.4) | 2 (1.4) | 3 (2.1) | 3 (2.1) |
| Deafness | 2 (1.4) | 2 (1.4) | 3 (2.1) | 1 (0.7) |
| Coronary stenosis | 2 (1.4) | 2 (1.4) | 2 (1.4) | 2 (1.4) |
| Coronary revascularization | 2 (1.4) | 2 (1.4) | 1 (0.7) | 1 (0.7) |
| Hepatoma | 2 (1.4) | 2 (1.4) | 1 (0.7) | 1 (0.7) |
| Arteriosclerosis obliterans | 2 (1.4) | 2 (1.4) | 0 (0.0) | 0 (0.0) |
| Lung cancer | 2 (1.4) | 2 (1.4) | 0 (0.0) | 0 (0.0) |
| Renal cancer | 2 (1.4) | 2 (1.4) | 0 (0.0) | 0 (0.0) |
| Herpes zoster | 2 (1.4) | 1 (0.7) | 2 (1.4) | 0 (0.0) |
| Asthma | 2 (1.4) | 1 (0.7) | 1 (0.7) | 0 (0.0) |
| Malaise | 2 (1.4) | 1 (0.7) | 1 (0.7) | 0 (0.0) |
| Urinary tract infection | 2 (1.4) | 1 (0.7) | 1 (0.7) | 0 (0.0) |
| Dyslipidemia | 2 (1.4) | 0 (0.0) | 5 (3.4) | 0 (0.0) |
| Anemia | 2 (1.4) | 0 (0.0) | 4 (2.8) | 0 (0.0) |
| Dermatitis | 2 (1.4) | 0 (0.0) | 1 (0.7) | 0 (0.0) |
| Diarrhea | 2 (1.4) | 0 (0.0) | 1 (0.7) | 0 (0.0) |
| Eczema | 2 (1.4) | 0 (0.0) | 1 (0.7) | 0 (0.0) |
| Helicobacter test positive | 2 (1.4) | 0 (0.0) | 1 (0.7) | 0 (0.0) |
| Muscle spasm | 2 (1.4) | 0 (0.0) | 1 (0.7) | 0 (0.0) |
| Reduction in blood pressure | 2 (1.4) | 0 (0.0) | 1 (0.7) | 0 (0.0) |
| Dysuria | 2 (1.4) | 0 (0.0) | 0 (0.0) | 0 (0.0) |
| Genital pruritus | 2 (1.4) | 0 (0.0) | 0 (0.0) | 0 (0.0) |
| Gastric cancer | 1 (0.7) | 1 (0.7) | 2 (1.4) | 2 (1.4) |
| Pyelonephritis | 1 (0.7) | 1 (0.7) | 2 (1.4) | 2 (1.4) |
| Atrial fibrillation | 1 (0.7) | 1 (0.7) | 2 (1.4) | 1 (0.7) |
| Burn injury | 1 (0.7) | 1 (0.7) | 2 (1.4) | 0 (0.0) |
| Breast cancer | 1 (0.7) | 1 (0.7) | 1 (0.7) | 1 (0.7) |
| Cellulitis | 1 (0.7) | 1 (0.7) | 1 (0.7) | 1 (0.7) |
| Epistaxis | 1 (0.7) | 1 (0.7) | 1 (0.7) | 1 (0.7) |
| Heart failure | 1 (0.7) | 1 (0.7) | 1 (0.7) | 1 (0.7) |
| Inguinal hernia | 1 (0.7) | 1 (0.7) | 1 (0.7) | 1 (0.7) |
| Tendon rupture | 1 (0.7) | 1 (0.7) | 1 (0.7) | 1 (0.7) |
| Contusion | 1 (0.7) | 1 (0.7) | 1 (0.7) | 0 (0.0) |
| Prostate cancer | 1 (0.7) | 1 (0.7) | 1 (0.7) | 0 (0.0) |
| B-cell lymphoma | 1 (0.7) | 1 (0.7) | 0 (0.0) | 0 (0.0) |
| Cholecystectomy | 1 (0.7) | 1 (0.7) | 0 (0.0) | 0 (0.0) |
| Colon cancer | 1 (0.7) | 1 (0.7) | 0 (0.0) | 0 (0.0) |
| Complete atrioventricular block | 1 (0.7) | 1 (0.7) | 0 (0.0) | 0 (0.0) |
| Decubitus ulcer | 1 (0.7) | 1 (0.7) | 0 (0.0) | 0 (0.0) |
| Diverticular hemorrhage | 1 (0.7) | 1 (0.7) | 0 (0.0) | 0 (0.0) |
| Enteritis | 1 (0.7) | 1 (0.7) | 0 (0.0) | 0 (0.0) |
| Hydrocephalus | 1 (0.7) | 1 (0.7) | 0 (0.0) | 0 (0.0) |
| Hysterectomy | 1 (0.7) | 1 (0.7) | 0 (0.0) | 0 (0.0) |
| Intracerebral hemorrhage | 1 (0.7) | 1 (0.7) | 0 (0.0) | 0 (0.0) |
| Malignant lymphoma | 1 (0.7) | 1 (0.7) | 0 (0.0) | 0 (0.0) |
| Parotid gland enlargement | 1 (0.7) | 1 (0.7) | 0 (0.0) | 0 (0.0) |
| Peripheral arterial occlusive disease | 1 (0.7) | 1 (0.7) | 0 (0.0) | 0 (0.0) |
| Premature ventricular contraction | 1 (0.7) | 1 (0.7) | 0 (0.0) | 0 (0.0) |
| Progressive supranuclear palsy | 1 (0.7) | 1 (0.7) | 0 (0.0) | 0 (0.0) |
| Renal abscess | 1 (0.7) | 1 (0.7) | 0 (0.0) | 0 (0.0) |
| Subarachnoid hemorrhage | 1 (0.7) | 1 (0.7) | 0 (0.0) | 0 (0.0) |
| Umbilical hernia | 1 (0.7) | 1 (0.7) | 0 (0.0) | 0 (0.0) |
| Urinary retention | 1 (0.7) | 1 (0.7) | 0 (0.0) | 0 (0.0) |
| Gastroenteritis | 1 (0.7) | 0 (0.0) | 4 (2.8) | 0 (0.0) |
| Spinal stenosis | 1 (0.7) | 0 (0.0) | 3 (2.1) | 2 (1.4) |
| Gastric ulcer | 1 (0.7) | 0 (0.0) | 3 (2.1) | 0 (0.0) |
| Hypertension | 1 (0.7) | 0 (0.0) | 3 (2.1) | 0 (0.0) |
| Coronary atherosclerosis | 1 (0.7) | 0 (0.0) | 2 (1.4) | 2 (1.4) |
| Allergic conjunctivitis | 1 (0.7) | 0 (0.0) | 2 (1.4) | 0 (0.0) |
| Edema | 1 (0.7) | 0 (0.0) | 2 (1.4) | 0 (0.0) |
| Chronic thyroiditis | 1 (0.7) | 0 (0.0) | 1 (0.7) | 0 (0.0) |
| Drug eruption | 1 (0.7) | 0 (0.0) | 1 (0.7) | 0 (0.0) |
| Periodontitis | 1 (0.7) | 0 (0.0) | 1 (0.7) | 0 (0.0) |
| Urolithiasis | 1 (0.7) | 0 (0.0) | 1 (0.7) | 0 (0.0) |
| Urticaria | 1 (0.7) | 0 (0.0) | 1 (0.7) | 0 (0.0) |
| Alopecia areata | 1 (0.7) | 0 (0.0) | 0 (0.0) | 0 (0.0) |
| Basedow's disease | 1 (0.7) | 0 (0.0) | 0 (0.0) | 0 (0.0) |
| Bladder hypertrophy | 1 (0.7) | 0 (0.0) | 0 (0.0) | 0 (0.0) |
| Dehydration | 1 (0.7) | 0 (0.0) | 0 (0.0) | 0 (0.0) |
| Erythema | 1 (0.7) | 0 (0.0) | 0 (0.0) | 0 (0.0) |
| Essential tremor | 1 (0.7) | 0 (0.0) | 0 (0.0) | 0 (0.0) |
| Fistula | 1 (0.7) | 0 (0.0) | 0 (0.0) | 0 (0.0) |
| Gait disturbance | 1 (0.7) | 0 (0.0) | 0 (0.0) | 0 (0.0) |
| Gallstone | 1 (0.7) | 0 (0.0) | 0 (0.0) | 0 (0.0) |
| Ganglion | 1 (0.7) | 0 (0.0) | 0 (0.0) | 0 (0.0) |
| Nausea | 1 (0.7) | 0 (0.0) | 0 (0.0) | 0 (0.0) |
| Numbness | 1 (0.7) | 0 (0.0) | 0 (0.0) | 0 (0.0) |
| Ocular hypertension | 1 (0.7) | 0 (0.0) | 0 (0.0) | 0 (0.0) |
| Oral discomfort | 1 (0.7) | 0 (0.0) | 0 (0.0) | 0 (0.0) |
| Peripheral neuritis | 1 (0.7) | 0 (0.0) | 0 (0.0) | 0 (0.0) |
| Rash | 1 (0.7) | 0 (0.0) | 0 (0.0) | 0 (0.0) |
| Renal disease | 1 (0.7) | 0 (0.0) | 0 (0.0) | 0 (0.0) |
| Retinal hemorrhage | 1 (0.7) | 0 (0.0) | 0 (0.0) | 0 (0.0) |
| Sleep disorder | 1 (0.7) | 0 (0.0) | 0 (0.0) | 0 (0.0) |
| Steatosis | 1 (0.7) | 0 (0.0) | 0 (0.0) | 0 (0.0) |
| Thyroid mass | 1 (0.7) | 0 (0.0) | 0 (0.0) | 0 (0.0) |
| Tooth extraction | 1 (0.7) | 0 (0.0) | 0 (0.0) | 0 (0.0) |
| Toothache | 1 (0.7) | 0 (0.0) | 0 (0.0) | 0 (0.0) |
| Verruca vulgaris | 1 (0.7) | 0 (0.0) | 0 (0.0) | 0 (0.0) |
| Vitreous floater | 1 (0.7) | 0 (0.0) | 0 (0.0) | 0 (0.0) |
| Osteoarthritis | 0 (0.0) | 0 (0.0) | 4 (2.8) | 2 (1.4) |
| Hepatic dysfunction | 0 (0.0) | 0 (0.0) | 3 (2.1) | 1 (0.7) |
| Diabetic neuropathy | 0 (0.0) | 0 (0.0) | 3 (2.1) | 0 (0.0) |
| Elevated blood pressure | 0 (0.0) | 0 (0.0) | 3 (2.1) | 0 (0.0) |
| Cerebral infarction | 0 (0.0) | 0 (0.0) | 2 (1.4) | 2 (1.4) |
| Cholangitis | 0 (0.0) | 0 (0.0) | 2 (1.4) | 1 (0.7) |
| Low back pain | 0 (0.0) | 0 (0.0) | 2 (1.4) | 1 (0.7) |
| Pneumonia | 0 (0.0) | 0 (0.0) | 2 (1.4) | 1 (0.7) |
| Dizziness | 0 (0.0) | 0 (0.0) | 2 (1.4) | 0 (0.0) |
| Elevated blood creatinine | 0 (0.0) | 0 (0.0) | 2 (1.4) | 0 (0.0) |
| Herpes simplex | 0 (0.0) | 0 (0.0) | 2 (1.4) | 0 (0.0) |
| Hyperuricemia | 0 (0.0) | 0 (0.0) | 2 (1.4) | 0 (0.0) |
| Sleep apnea syndrome | 0 (0.0) | 0 (0.0) | 2 (1.4) | 0 (0.0) |
| Angina | 0 (0.0) | 0 (0.0) | 1 (0.7) | 1 (0.7) |
| Cervical cancer | 0 (0.0) | 0 (0.0) | 1 (0.7) | 1 (0.7) |
| Cryptogenic death | 0 (0.0) | 0 (0.0) | 1 (0.7) | 1 (0.7) |
| Disc herniation | 0 (0.0) | 0 (0.0) | 1 (0.7) | 1 (0.7) |
| Extramammary Paget's disease | 0 (0.0) | 0 (0.0) | 1 (0.7) | 1 (0.7) |
| Ileus | 0 (0.0) | 0 (0.0) | 1 (0.7) | 1 (0.7) |
| Infection at implantation site | 0 (0.0) | 0 (0.0) | 1 (0.7) | 1 (0.7) |
| Loss of consciousness | 0 (0.0) | 0 (0.0) | 1 (0.7) | 1 (0.7) |
| Ossification of ligament and spine | 0 (0.0) | 0 (0.0) | 1 (0.7) | 1 (0.7) |
| Pheochromocytoma | 0 (0.0) | 0 (0.0) | 1 (0.7) | 1 (0.7) |
| Prostatitis | 0 (0.0) | 0 (0.0) | 1 (0.7) | 1 (0.7) |
| Renal failure | 0 (0.0) | 0 (0.0) | 1 (0.7) | 1 (0.7) |
| Retinal detachment | 0 (0.0) | 0 (0.0) | 1 (0.7) | 1 (0.7) |
| Spinal injury | 0 (0.0) | 0 (0.0) | 1 (0.7) | 1 (0.7) |
| Abnormal value in prostate examination | 0 (0.0) | 0 (0.0) | 1 (0.7) | 0 (0.0) |
| Aortic stenosis | 0 (0.0) | 0 (0.0) | 1 (0.7) | 0 (0.0) |
| Dermal cyst | 0 (0.0) | 0 (0.0) | 1 (0.7) | 0 (0.0) |
| Diabetic nephropathy | 0 (0.0) | 0 (0.0) | 1 (0.7) | 0 (0.0) |
| Eccrine poroma | 0 (0.0) | 0 (0.0) | 1 (0.7) | 0 (0.0) |
| Enteritis | 0 (0.0) | 0 (0.0) | 1 (0.7) | 0 (0.0) |
| Exertional dyspnea | 0 (0.0) | 0 (0.0) | 1 (0.7) | 0 (0.0) |
| Hand-foot-and-mouth disease | 0 (0.0) | 0 (0.0) | 1 (0.7) | 0 (0.0) |
| Hyperammonemia | 0 (0.0) | 0 (0.0) | 1 (0.7) | 0 (0.0) |
| Hypercalcemia | 0 (0.0) | 0 (0.0) | 1 (0.7) | 0 (0.0) |
| Hypozincemia | 0 (0.0) | 0 (0.0) | 1 (0.7) | 0 (0.0) |
| Insomnia | 0 (0.0) | 0 (0.0) | 1 (0.7) | 0 (0.0) |
| Joint swelling | 0 (0.0) | 0 (0.0) | 1 (0.7) | 0 (0.0) |
| Lacunar infarct | 0 (0.0) | 0 (0.0) | 1 (0.7) | 0 (0.0) |
| Ligament injury | 0 (0.0) | 0 (0.0) | 1 (0.7) | 0 (0.0) |
| Ligamentitis | 0 (0.0) | 0 (0.0) | 1 (0.7) | 0 (0.0) |
| Loose stool | 0 (0.0) | 0 (0.0) | 1 (0.7) | 0 (0.0) |
| Mediastinal cyst | 0 (0.0) | 0 (0.0) | 1 (0.7) | 0 (0.0) |
| Nephrolithiasis | 0 (0.0) | 0 (0.0) | 1 (0.7) | 0 (0.0) |
| Neurogenic bladder | 0 (0.0) | 0 (0.0) | 1 (0.7) | 0 (0.0) |
| Obstructive pulmonary disease | 0 (0.0) | 0 (0.0) | 1 (0.7) | 0 (0.0) |
| Otitis media | 0 (0.0) | 0 (0.0) | 1 (0.7) | 0 (0.0) |
| Pain | 0 (0.0) | 0 (0.0) | 1 (0.7) | 0 (0.0) |
| Palpitation | 0 (0.0) | 0 (0.0) | 1 (0.7) | 0 (0.0) |
| Periarthritis | 0 (0.0) | 0 (0.0) | 1 (0.7) | 0 (0.0) |
| Phlebitis | 0 (0.0) | 0 (0.0) | 1 (0.7) | 0 (0.0) |
| Piriformis muscle syndrome | 0 (0.0) | 0 (0.0) | 1 (0.7) | 0 (0.0) |
| Primary aldosteronism | 0 (0.0) | 0 (0.0) | 1 (0.7) | 0 (0.0) |
| Prostatic hyperplasia | 0 (0.0) | 0 (0.0) | 1 (0.7) | 0 (0.0) |
| Purulence | 0 (0.0) | 0 (0.0) | 1 (0.7) | 0 (0.0) |
| Rheumatoid arthritis | 0 (0.0) | 0 (0.0) | 1 (0.7) | 0 (0.0) |
| Skin rash | 0 (0.0) | 0 (0.0) | 1 (0.7) | 0 (0.0) |
| Urethritis | 0 (0.0) | 0 (0.0) | 1 (0.7) | 0 (0.0) |
| Venous thrombosis | 0 (0.0) | 0 (0.0) | 1 (0.7) | 0 (0.0) |
| Wound | 0 (0.0) | 0 (0.0) | 1 (0.7) | 0 (0.0) |
